# Of mice, flies – and men? Comparing fungal infection models for large-scale screening efforts

**DOI:** 10.1242/dmm.019901

**Published:** 2015-05-01

**Authors:** Sascha Brunke, Jessica Quintin, Lydia Kasper, Ilse D. Jacobsen, Martin E. Richter, Ekkehard Hiller, Tobias Schwarzmüller, Christophe d'Enfert, Karl Kuchler, Steffen Rupp, Bernhard Hube, Dominique Ferrandon

**Affiliations:** ^1^ Integrated Research and Treatment Center, Center for Sepsis Control and Care (CSCC), University Hospital, 07747 Jena, Germany; ^2^ Department of Microbial Pathogenicity Mechanisms, Hans Knöll Institute, 07745 Jena, Germany; ^3^ Equipe Fondation Recherche Médicale, Unité Propre de Recherche 9022 du Centre National de la Recherche Scientifique (CNRS), Institut de Biologie Moléculaire et Cellulaire (IBMC), Université de Strasbourg, 67084 Strasbourg, France; ^4^ Research Group Microbial Immunology, Hans Knöll Institute, 07745 Jena, Germany; ^5^ Friedrich Schiller University, 07743 Jena, Germany; ^6^ Institute for Clinical Chemistry and Laboratory Medicine, Jena University Hospital, 07747 Jena, Germany; ^7^ Department of Molecular Biotechnology, Fraunhofer Institute for Interfacial Engineering and Biotechnology, 70569 Stuttgart, Germany; ^8^ Department of Medical Biochemistry, Max F. Perutz Laboratories, Medical University Vienna, 1030 Vienna, Austria; ^9^ Institut Pasteur, Unité Biologie et Pathogénicité Fongiques, Département Génomes et Génétique, 75015 Paris, France; ^10^ INRA, USC2019, 75015 Paris, France

**Keywords:** *Candida glabrata*, Mutant library, *Drosophila melanogaster*, Alternative infection models, Signature-tagged mutagenesis, Fungal virulence factors

## Abstract

Studying infectious diseases requires suitable hosts for experimental *in vivo* infections. Recent years have seen the advent of many alternatives to murine infection models. However, the use of non-mammalian models is still controversial because it is often unclear how well findings from these systems predict virulence potential in humans or other mammals. Here, we compare the commonly used models, fruit fly and mouse (representing invertebrate and mammalian hosts), for their similarities and degree of correlation upon infection with a library of mutants of an important fungal pathogen, the yeast *Candida glabrata*. Using two indices, for fly survival time and for mouse fungal burden in specific organs, we show a good agreement between the models. We provide a suitable predictive model for estimating the virulence potential of *C. glabrata* mutants in the mouse from fly survival data. As examples, we found cell wall integrity mutants attenuated in flies, and mutants of a MAP kinase pathway had defective virulence in flies and reduced relative pathogen fitness in mice. In addition, mutants with strongly reduced *in vitro* growth generally, but not always, had reduced virulence in flies. Overall, we demonstrate that surveying *Drosophila* survival after infection is a suitable model to predict the outcome of murine infections, especially for severely attenuated *C. glabrata* mutants. Pre-screening of mutants in an invertebrate *Drosophila* model can, thus, provide a good estimate of the probability of finding a strain with reduced microbial burden in the mouse host.

## Abstract (precis)

**Summary:** Can the fitness of deletion mutants in a murine model be predicted by their virulence in *Drosophila melanogaster*? For a fungal pathogen, the answer is, mostly, yes.

## INTRODUCTION

The selection of suitable models is crucial in infection biology research. Deciding on the right model system for the biological question at hand requires deliberate weighing of many parameters, such as cost, amount of labor involved, throughput rate, degree of similarity to the human host, and ethical considerations. For example, the use of comparatively simple *in vitro* models when screening for novel antimicrobial drug candidates and investigating putative virulence factors in microbial pathogens is well established. However, *in vivo* models are still indispensable to provide the link between a gene and the clinically relevant outcome – disease or death of the host. Many different models – vertebrate and invertebrate – have been described in the past for the investigation of microbial virulence. Generally, using a murine model is considered the gold standard for most infections, due to its comparably high similarity to humans in terms of metabolism, body temperature, and immune system functions. Yet, working with mice requires specialized personnel, poses many practical difficulties, is often expensive and time consuming, and requires specific ethical considerations. Alternative *in vivo* infection models are, therefore, used ever more frequently. For pathogenic fungi, for example, these include – in no specific order: the vertebrate zebrafish model (Chao et al., 2010; Tobin et al., 2012), the embryonated chicken egg model (Jacobsen et al., 2011, 2010b), the nematode *Caenorhabditis elegans* (Breger et al., 2007), the insect models *Galleria mellonella* (Cotter et al., 2000; Jacobsen, 2014) and *Drosophila melanogaster* (Glittenberg et al., 2011; Limmer et al., 2011; Roetzer et al., 2008), and some more specialized models like *Acanthamoeba* spp. or *Dictyostelium discoideum* (Mylonakis et al., 2007; Steenbergen et al., 2001). Especially for large-scale screening efforts, for example, with libraries of hundreds or thousands of mutants, the benefits of using these systems are evident: they are generally easier to handle and, in contrast to mice, the use of hundreds of flies or worms for infection experiments is considered ethically acceptable.

Unfortunately, limitations remain associated with these non-mammalian infection systems. First and foremost, the body temperature of the non-mammalian hosts is generally significantly lower than that of humans. Indeed, many microbial virulence factors are expressed only, or more readily, at human body temperature. An important example is the morphological transition between yeasts and hyphae by *Candida albicans*, which is generally considered essential for full virulence of this fungus (Jacobsen et al., 2012) and which is induced by growth at 37°C. However, hyphae formation of *C. albicans* can also occur at lower temperatures (25-28°C) in *in vivo* systems such as zebrafish (Brothers et al., 2011) or *C. elegans* (Pukkila-Worley et al., 2009), and plays a crucial role in pathogenesis in these systems. This indicates that additional host-related factors or conditions can supersede the need for increased temperature in these models. Furthermore, the ability to grow at 37°C itself can be considered a virulence factor of its own in pathogenic fungi (Casadevall, 2005) and other microbes. For example, the calcineurin pathway is required both for full virulence, and for growth at temperatures of 37°C and above in *C. glabrata* (Chen et al., 2012) and *Cryptococcus neoformans* (Odom et al., 1997). To test these temperature-dependent aspects of pathogenesis, the suitability of many alternative infection models is limited, with few exceptions, such as *G. mellonella*, which withstands temperatures up to 37°C (Desalermos et al., 2012).

**TRANSLATIONAL IMPACT**

**Clinical issue**

Understanding mechanisms of infectious diseases requires suitable animal models for replicating human infections *in vivo*. Mammals such as mice are generally the model of choice because they closely resemble humans in many – although not all – biologically relevant aspects, like general anatomy and immune functions. However, for ethical and practical reasons, alternative models, ranging from vertebrates (like zebrafish) to insects or even amoebae, are increasingly used. Obviously, these models deviate substantially from humans. Hence, the use of alternative infection models is accompanied by an ongoing debate about how well these models reflect disease in mammals and, ultimately, humans.

**Results**

This work establishes a systemic infection model of mice for the simultaneous investigation of large sets of mutant strains of the human pathogenic fungus *Candida glabrata*, and an individual infection screen for hundreds of fungal mutants in fruit flies. When comparing these models, the relative fitness of mutants of *C. glabrata* in murine infections was predicted by using an alternative *Drosophila melanogaster* infection system. Moreover, fly mortality predicts growth of fungal mutant strains in mouse organs better than *in vitro* pre-screens, indicating similar infection-specific functions of many fungal genes in both models. Finally, several genes of related functional classes were found to be relevant in both models, although, interestingly, some seemed to be specific for each infection model.

**Implications and future directions**

This mutant library screen is one of the largest to date to compare a vertebrate and an invertebrate infection model. The data obtained are especially relevant for future large-scale mutant library screens of pathogenic microbes: a broad screen in an invertebrate host can pre-select conspicuous mutants to be validated and investigated in-depth in the ethically and practically more-challenging mouse models. This will allow researchers to better judge the applicability of data from alternative models, and help reduce and refine the use of mice in infection biology research.

In addition, although the evolutionary ancient Toll pathway shares many similarities with its counterpart in mammalian innate immune recognition pathways (Ferrandon et al., 2007), the immune systems of humans and insects differ fundamentally in many aspects. Certain fungal immune evasion factors are, therefore, likely to escape detection in mutant screens using these models. Mammalian and other vertebrate hosts, such as zebrafish (Gratacap and Wheeler, 2014; Tobin et al., 2012), are better suited in this respect. In these models, many additional immune responses are conserved in comparison to humans, from an even more similar innate immunity to the adaptive immune response in mice and (adult) zebrafish. Yet, even these models do not perfectly simulate human infections in all aspects. For example, the fungal pathogen *C. glabrata* is virtually non-pathogenic in most systemic infection models of mice, both immunosuppressed and immunocompetent (Brieland et al., 2001; Jacobsen et al., 2010a), betraying its high mortality in humans. Yet, *C. glabrata* easily persists for weeks even in fully immunocompetent animals and can be re-isolated in high numbers from their organs (Jacobsen et al., 2010a). The method of choice to estimate the fitness in the host and, hence, the virulence potential in a murine model is, therefore, re-isolation and determination of colony-forming units (Jacobsen et al., 2010a). For *Candida glabrata*, one main difference between humans and mice is that the latter are not normally colonized by the fungus in its commensal stage. This, and the fact that susceptibility to *C. glabrata* increases with age in humans (Arendrup, 2010), cannot be fully mirrored with current models. Nonetheless, murine models have proven highly useful for the investigation of fungal infections. Mice have been used in the past for large-scale screenings, often with a pool of signature-tagged mutants to minimize the number of animals required. Mutant libraries of *C. neoformans* (Liu et al., 2008), *C. albicans* (Noble et al., 2010) and *Aspergillus fumigatus* (Brown et al., 2000) have been successfully screened this way. Some smaller screening efforts have also taken place with *D. melanogaster*, for example, with a set of *C. albicans* transcription factor mutants (Chamilos et al., 2009).

Despite the differences between insects and mammals, the fruit fly is considered a suitable alternative model to detect virulence factors of microbes. The relative ease of handling and the aforementioned ethical considerations outweigh many of the inherent limitations of the model. In addition, fruit flies present a complex organism in which infections are not limited to the intestine (like in *C. elegans*), and the innate immune system of *Drosophila*, based on both cellular and humoral responses, is rather well understood (Ferrandon et al., 2007). This is flanked by sophisticated genetics. In *Drosophila*, host survival rates can be used as a measure of virulence. Depending on the infecting fungus, flies must be suppressed in their natural immunity to fungal infections (Quintin et al., 2013) or can be fully immuno-competent (Glittenberg et al., 2011). The most commonly used fly strains are *D. melanogaster* deficient in Toll pathway signaling, which are also susceptible to *C. glabrata.* Like mice, wild-type flies are resistant to *C. glabrata* infections (Quintin et al., 2013; Roetzer et al., 2008), although they succumb to some degree to *C. albicans* infections (Glittenberg et al., 2011). In both mice and Toll pathway mutant flies, the cellular arm of host defense plays a paramount role.

In this work, we investigated whether the simple and facile *D. melanogaster* model can predict the outcome of infections in the more complex mouse host. As a model for a human pathogen, we used a large collection of *C. glabrata* mutants (Schwarzmüller et al., 2014). *C. glabrata* is one of the most important fungal pathogens in the clinical setting with a mortality often in excess of 50% (Perlroth et al., 2007). All in all, *Candida* species account for about 10% of all bloodstream infections (Liu et al., 2010), with an even higher rate in intensive care unit patients (Gullo, 2009). We determined the survival rate of fruit flies after infection with *C. glabrata* mutants and, additionally, the fungal fitness in mice by systemic infections with mutant pools. We determined whether genes required for fitness and/or virulence are common to these two models or specific to either one. We show that virulence in the simple host *D. melanogaster* predicts fitness in mice much better than the *in vitro* growth rates of mutants.

## RESULTS

To compare the *Drosophila melanogaster* and *Mus musculus* microbial infection models, we used a recently created large-scale library of *C. glabrata* gene-deletion mutants, enriched for processes that are likely to be involved in pathogenesis or transcriptional regulation (Schwarzmüller et al., 2014). The use of hundreds of mutants instead of a few selected strains allowed a thorough comparison of these models in order to determine whether the fruit fly model can serve to predict fitness and virulence potential in the mouse. Of the library, 416 individual *C. glabrata* mutants were first tested in *D. melanogaster*, and 114 strains were then selected to be tested in murine pool infections. The strains were chosen to represent all strongly attenuated and hypervirulent mutants from the fly model, and a random set of mutants with wild-type-like virulence in flies.

### The *D. melanogaster* model

**Fig. 1.**
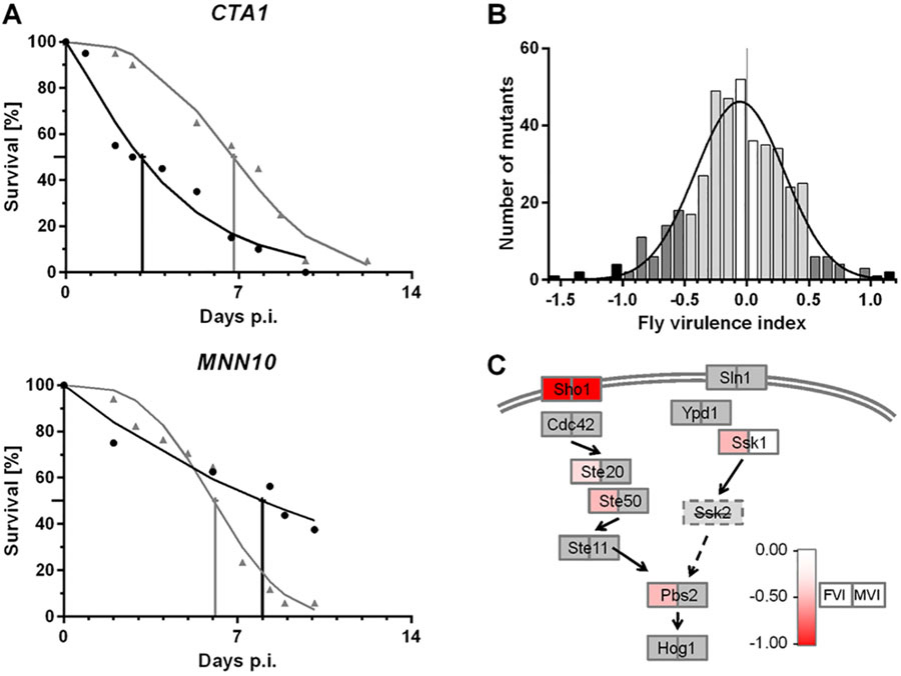
**Fly virulence index (FVI) of 428 *C. glabrata* mutants.** (A) Data fitting for FVI calculation is shown in two examples (*CTA1*- and *MNN10*-deletion mutants). The calculation was based on a Weibull distribution fit to the fly survival data. For each experimental group, the LT_50_ ratio of mutant to wild type was calculated. Black circles, measured fly survival for mutant strain, gray triangles, corresponding wild-type data; solid graph lines, curves calculated by Weibull formula; vertical lines, LT_50_ values. (B) Distribution of the FVI. In the mean, mutants were slightly attenuated in the fly, with a Gaussian fit mean of −0.074. White and light gray, neutral mutants, gray, increased or decreased virulence, black, highly increased or decreased virulence. (C) Deletions of HOG pathway genes lead to reduced virulence in flies. All tested deletion mutants had FVI values of −0.36 or less (see color scale; gray indicates no data). A similar reduction in virulence was obtained in mice (MVI) for deletion of *SHO1* but not of *SSK1*. Note that the Sln1 arm of the pathway is disrupted due to a non-functional Ssk2 in *C. glabrata* ATCC 2001. Modified from Gregori et al. (2007).

We started by testing the ∼400 *C. glabrata* mutants of the gene deletion collection by using our immunodeficient (Toll-signaling-defective; *MyD88*) *D. melanogaster* model (Quintin et al., 2013). The biologically relevant outcome in this model was the death of the host flies, which was recorded on a daily basis. We used the Weibull distribution originally suggested by Glittenberg et al. for *C. albicans* (Glittenberg et al., 2011) to fit the Kaplan–Meier survival plots obtained by these experiments (Fig. 1A). As a measure for virulence, we calculated a fly virulence index (FVI) of all mutants (supplementary material Table S1). This was done by determining the time (LT_50_) at which 50% of *D. melanogaster* had died according to the model. The log_2_ ratios of these LT_50_ values were calculated between flies infected with the deletion mutant and those infected with wild-type *C. glabrata* in the same infection group (see Materials and Methods for a more detailed description).

Most mutants did not deviate much from the wild type in their virulence when using this measure, as would be expected from a large collection of single-gene deletions. A Gaussian distribution fit for the FVI data has a mean of −0.074, corresponding to a 5% increase in LT_50_ when using the mutants compared with the wild type (Fig. 1B). We set the FVI cut-off for changes signifying an increased or decreased virulence in the fly to ±0.5 (1.4-fold), which we considered potentially biologically relevant. Altogether, 58 mutants (13.5%) were classified hypovirulent, and 23 mutants (5.4%) were hypervirulent. These comparatively high values were not unexpected, as the original mutant collection was already enriched for mutants with potential virulence defects (Schwarzmüller et al., 2014). Furthermore, nine of these mutants (2.1%) could be classified as strongly hypovirulent (FVI<−1.0) and three (0.7%) as strongly hypervirulent (FVI>1.0).

We used previously published *in vitro* growth data of these mutants to expand our analysis. In this context, reduced growth was defined as a >2σ increase in generation time in complex medium compared with the mean of all strains (Schwarzmüller et al., 2014). The percentage of mutants with reduced *in vitro* growth was similar to the proportion of hypovirulent strains by FVI values (13.7% vs. 13.5%). Similarly, 6.5% of mutants showed increased (>2σ) growth *in vitro*, compared with 5.4% that were hypervirulent in flies. There is some correlation (*r*=0.43) of the fly virulence index to the relative growth rate of the mutants *in vitro* (Schwarzmüller et al., 2014), but many of the most strongly attenuated mutants *in vivo* still showed normal *in vitro* growth rates (see below).

A gene ontology (GO) slim-term enrichment analysis of the sets of mutants that were attenuated in *Drosophila* virulence revealed an over-representation of biological processes related to the response to stimuli and signal transduction – but also maintenance of homeostasis – in the hypovirulent mutants, even after taking the library-creation bias into account (Table 1). No significant GO term enrichment was found for the hypervirulent mutants.

**Table 1.**
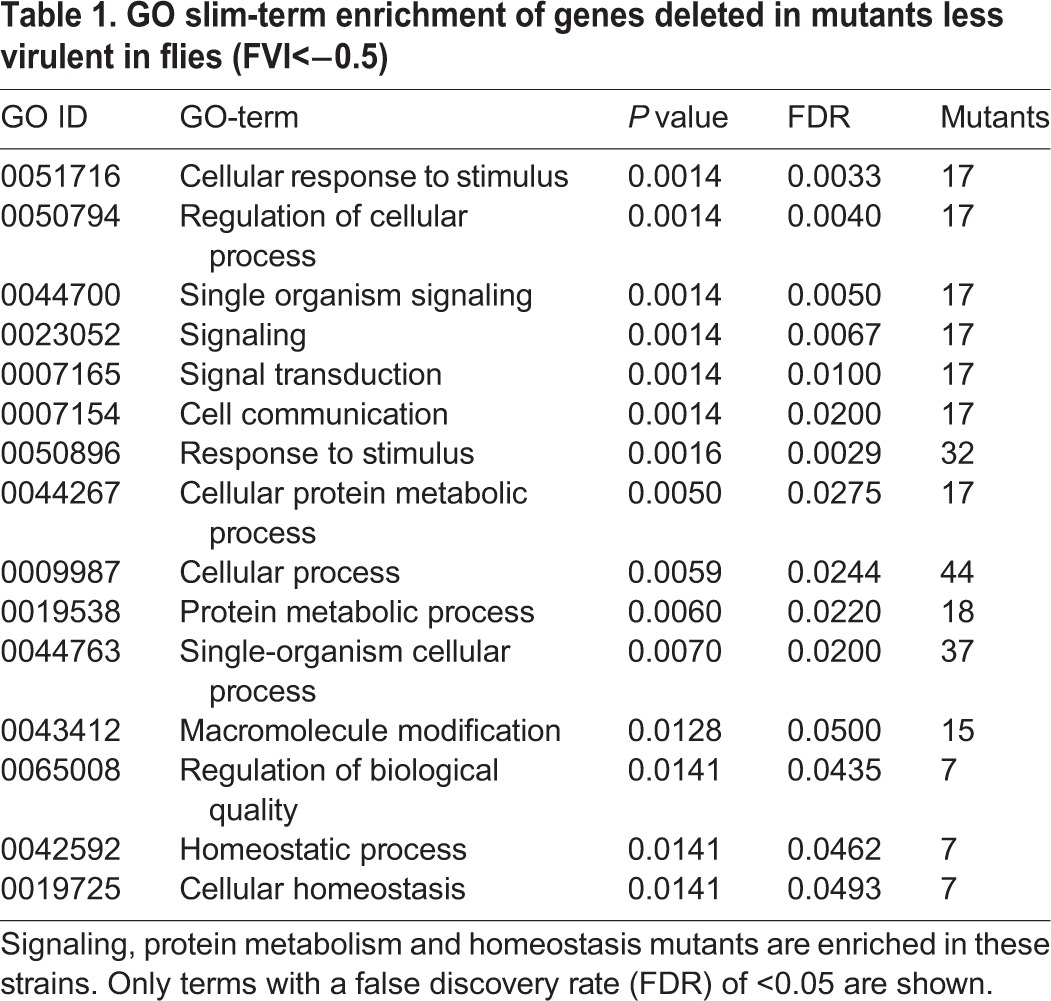
**GO slim-term enrichment of genes deleted in mutants less virulent in flies (FVI<−0.5)**

Genes important for cell wall integrity scored among the most strongly attenuated in our *Drosophila* model (Table 2, supplementary material Table S1). Deletion of the homologs of *SSD1* (with normal *in vitro* growth) and its regulator *CBK1* (with reduced growth), representing the cell wall integrity part of the RAM network (Saputo et al., 2012), led to strongly hypovirulent mutants (FVI<−1.0). Similarly, deletions of genes involved in the Slt2/Mpk1 MAPK cascade for cell wall biogenesis (*ROM2*, *PKH1*, *PKH2*, *YPK2*, *MKK2* and *RLM1*) are all hypovirulent or strongly hypovirulent without any measurable defect in *in vitro* growth. Deleting the main chitin synthase gene *CHS3* of the cell wall or a gene encoding a part of its Golgi-to-membrane exomere transport complex (*CHS5*) also led to hypovirulent *C. glabrata* mutants in our system. Finally, disturbing the glucan part of cell wall biogenesis also appeared to reduce virulence in flies: deletion mutants of the glucan synthase genes *FKS2* and *FKS3* both had FVI values of less than −0.5 (*fks1*Δ was not part of the mutant collection). None of these were defective in *in vitro* growth. *MNN10*, as a gene encoding a mannosyltransferase with function in barrier septum formation, was required for full fly virulence (mutant FVI of −0.54) but also for normal *in vitro* growth. Strains lacking the aspartic proteases Yps7 and Yps10 were also decreased in virulence in the fly model, an observation reminiscent of our recent finding that a *yps1-11*Δ strain lacks virulence in the *Drosophila* model (Quintin et al., 2013). Yps7 has recently been associated with cell wall integrity (Bairwa et al., 2014). In addition, both Yps7 and Yps10 also have a role in macrophage survival and murine virulence, as shown by Kaur et al. (2007).

**Table 2.**
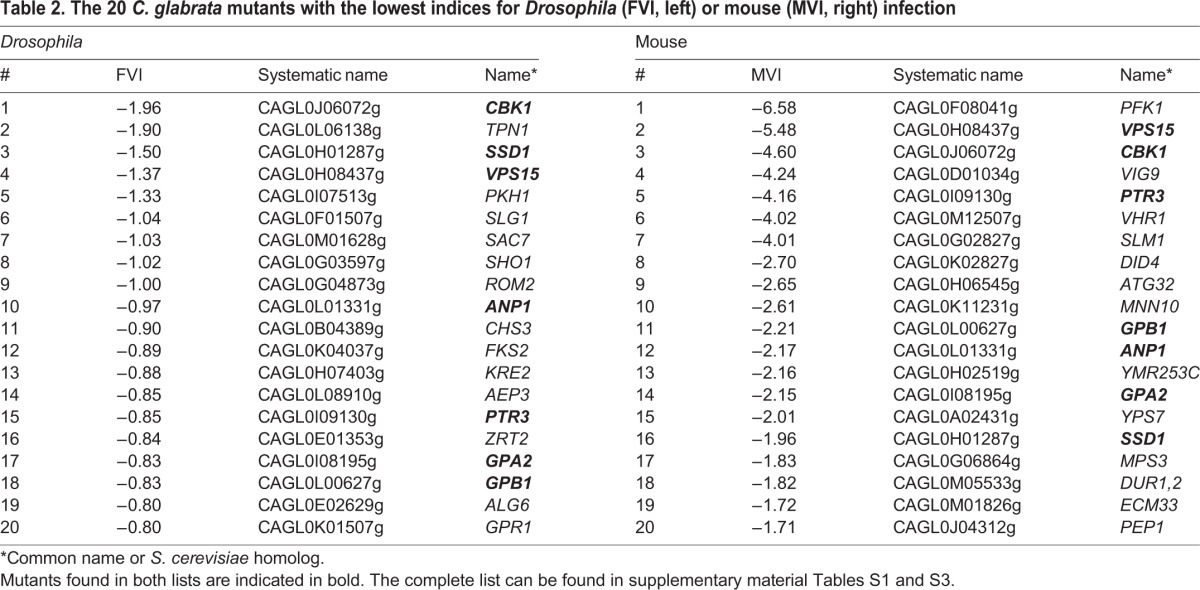
**The 20 *C. glabrata* mutants with the lowest indices for *Drosophila* (FVI, left) or mouse (MVI, right) infection**

Interestingly, a deletion mutant of the high-osmolarity glycerol (HOG) pathway gene *SHO1* exhibited a severely decreased FVI (−1.02) in our library strains, in agreement with a previous *Drosophila* screen in which an independent mutant was used in the same ATCC 2001 background (Quintin et al., 2013). Deleting *PBS2*, which encodes a MAPK kinase situated downstream of Sho1 in HOG signaling, also led to a markedly reduced FVI (−0.50). Similar values were obtained for the other HOG pathway mutants, *ste20*Δ (−0.36) and *ste50*Δ (−0.50), showing that FVI data of mutants along a pathway are in good agreement (Fig. 1C). Finally, a deletion mutant of the putative phosphofructokinase *PFK1* is strongly reduced in virulence, which is probably due to its generic *in vitro* growth defect (Schwarzmüller et al., 2014).

The most hypervirulent mutant in our *Drosophila* screen was of a homolog of *S. cerevisiae CTR2*, a high-affinity Cu^2+^ transporter. Deletion of this gene in yeast is known to decrease the sensitivity to otherwise toxic amounts of Cu^2+^ (Kampfenkel et al., 1995). Surprisingly, the catalase deletion mutant *cta1*Δ was also hypervirulent in our model (Fig. 1A). Overall, cell wall integrity pathways seemed to be paramount for *C. glabrata* virulence during *Drosophila* infections, as were selected genes for growth and immune cell interaction.

### The murine pool model

**Fig. 2.**
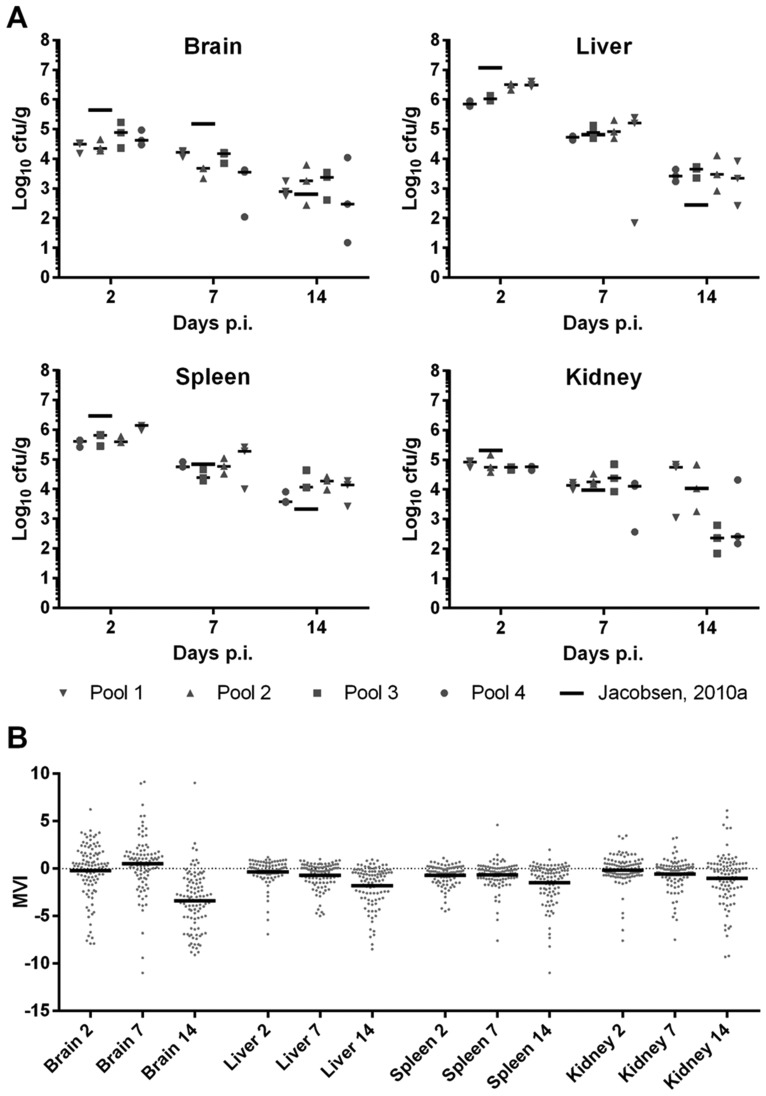
**Fungal burdens in different organs.** (A) Absolute fungal burden and decrease during the infection experiment were comparable among the four pools, and to the data obtained from a single-strain infection experiment using the same model (Jacobsen et al., 2010a). Black bars show the median values. (B) Enrichment or depletion of mutants in the organs. Dots represent MVIs of all individual mutants retrieved from mice. Negative MVIs indicate depletion of the fungal load of a mutant relative to the wild type. Black bars indicate mean MVI. The mutants are generally less fit in the mouse organs than the wild type, and their depletion generally increases continuously over time (with the exception of the brain).

We devised a mutant pool approach to test the *C. glabrata* strains. In our systemic mouse infection model, animals do not succumb to *C. glabrata* infection, which allows for long-term experiments to observe competition of mutants within a pool. Four pools were set up with approximately 40 mutants each (supplementary material Table S2), representing hyper- and hypovirulent mutants as well as mutants with normal virulence in the fly model. Assignment to each pool was random for all mutants. After tail-vein infection of mice with different pools of *C. glabrata*, all animals remained clinically healthy, comparable to single-strain challenge experiments (Jacobsen et al., 2010a). The fungal burden in the organs at the three sampling days (Fig. 2A) were also in very good agreement with our previous single-strain data (Jacobsen et al., 2010a), indicating a normal progression of disease in the animals.

As a biological meaningful measure, we calculated the depletion or enrichment of mutants relative to wild type after re-isolation. This was done by dividing their respective re-isolation-to-infection ratios (the mouse virulence index, or MVI; see Materials and Methods for details). At any given time point in any organ, the mean virulence indices of the mutants were found to be slightly lower than those of the wild type, indicating – as expected and similar to the fly model – that, whereas most individual mutants did not deviate much in fitness from the wild type, on average, gene deletions result in at least slight growth defects in the murine system (Fig. 2B). Over time, this tendency increased in all organs, with the exception of the brain, where the distribution of mutants fluctuated strongly. The latter was probably due to the low re-isolation frequency in brain, as it favors random fluctuations and ‘founder effects’ over actual selection pressure.

**Fig. 3.**
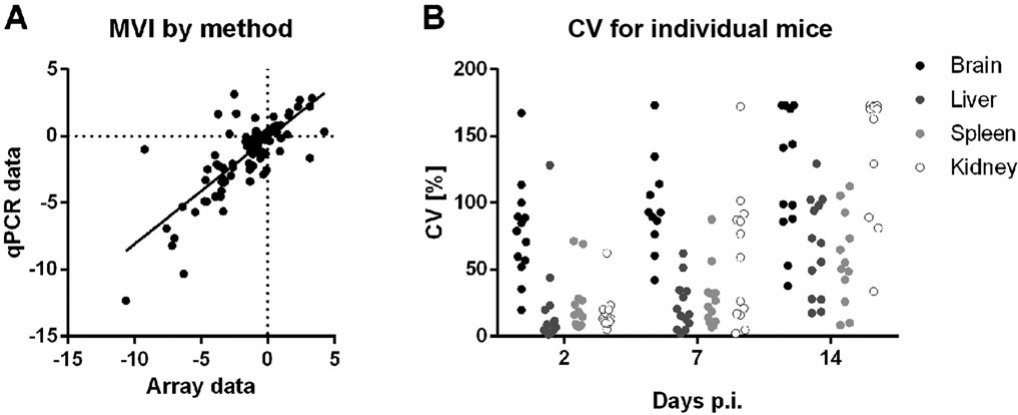
**Individual barcode detection by qPCR.** (A) Comparison of microarray and qPCR to detect barcodes from organ samples shows good agreement. MVI of selected mutants were calculated based on either the microarray data from pooled fungal gDNA of three mice, or the mean of the qPCR data from the three individual mice samples. Each dot represents the MVIs calculated for one strain at a single time point by microarray and qPCR (see supplementary material Table S2 for the list of genes). (B) The coefficient of variation (CV) increases over time between individual mice. The CV for each mutant (represented by a single point; same genes as for panel A) was calculated from the qPCR data of different mice. An increase in variation can be observed over time, and the brain exhibits a generally high CV at each time point.

Using microarray analysis, we were able to detect most of the barcodes of the injected strains in all infected organs (supplementary material Table S3). For our analysis, we have used combined DNA of pools isolated from the same organ of three individual mice at each time-point. In order to validate this detection approach, we compared array data of selected mutants with quantitative PCR (qPCR) data obtained from the same samples isolated from the individual mice. The overall correlation between the array-based MVI and the means of the qPCR data was good (Pearson *r*=0.78, Fig. 3A) and in range of similar comparisons using expression data (Morey et al., 2006), with the exception of the kidney (liver *r*=0.96, spleen *r*=0.90, brain *r*=0.87, kidney *r*=0.44).

To further study the inter-replicate variation, we investigated the variation of the barcode signals of selected mutants between individual mice. We focused on mutants representing examples for both well-correlating, and differing FVI and MVI values. As a measure of scatter among individual mice, we calculated the coefficient of variation (CV), i.e. the standard deviation of the mean from qPCR measurements of individual organs in percent. As shown in Fig. 3B, the CV generally increased over time, indicating an increasing effect of random or individual and specific processes on the relative frequency of the mutants. Similarly, the frequency of mutant re-isolation from the brain shows a very high inter-individual CV already at day 2 post infection (p.i.), again hinting at a dominance of random events over genetic determinants in the colonization of this organ by *C. glabrata*. At day 7, the organs with the smallest differences between individual mice were the liver and the spleen. Given all these observations and the low colony forming unit (cfu) counts from the brain, we decided to exclude the brain from our further analysis, and defined the total MVI as the mean value over all time points obtained from liver, spleen and kidney.

Based on the total MVI of individual genes, the most strongly depleted mutants were deletion strains of: *PFK1* – similar to the fly model; a kinase involved in vacuolar protein sorting (*VPS15*); the RAM network kinase gene *CBK1* – again similar to the fly model; *PTR3*, probably encoding part of the sensor complex for external amino acids; the pseudogene CAGL0M12507g, which is homologous to the *S. cerevisiae* vitamin H transporter transcriptional activator gene (*VHR1*); and *SLM1*, probably encoding a downstream effector of the TOR pathway. Furthermore, deletion of *DID4*, whose homolog is also involved in vacuolar protein sorting, the mitophagy-related gene *ATG32*, and the genes involved in protein mannosylation, *MNN10* and *VIG9*, led to a strong reduction in overall fungal burden. Of these ten deletion mutants, five also had moderate (*VPS15*) to severe (*PFK1*, *CBK1*, *VIG9*, *MNN10*) *in vitro* growth defects, whereas – in contrast – one mutant (*DID4*) displayed an increased *in vitro* growth rate. Overall, there is only some correlation (*r*=0.31, *P*<0.01) of the MVI with the published *in vitro* fitness of the mutants (Schwarzmüller et al., 2014; supplementary material Fig. S1). Further attenuated mutants included, among others, *yps5*Δ (CAGL0E01771g) and *yps7*Δ, and – like in *D. melanogaster* – deletion mutants of genes involved in cell wall biosynthesis and maintenance (*ECM33*, *CHS1*), and protein glycosylation (*ANP1*).

Among the ten mutants enriched in the organs (mean MVI>0.5) were *TIP41*, an ortholog of a negative regulator gene of the TOR pathway (confirming the importance of the TOR pathway that was implied by attenuation of mutants such as *slm1*Δ), *CKB2*, encoding the inhibitory subunit of the protein kinase CK2 in *S. cerevisiae*, and *GPR1*, which in baker's yeast is involved in glucose detection. The *AXL2* and *BNI1* genes, both involved in axial bud site selection, also showed increased MVIs. In contrast to the depleted mutants with low MVIs, however, these enrichments were largely represented by a single organ, the kidney.

### Correlation between the models

**Fig. 4.**
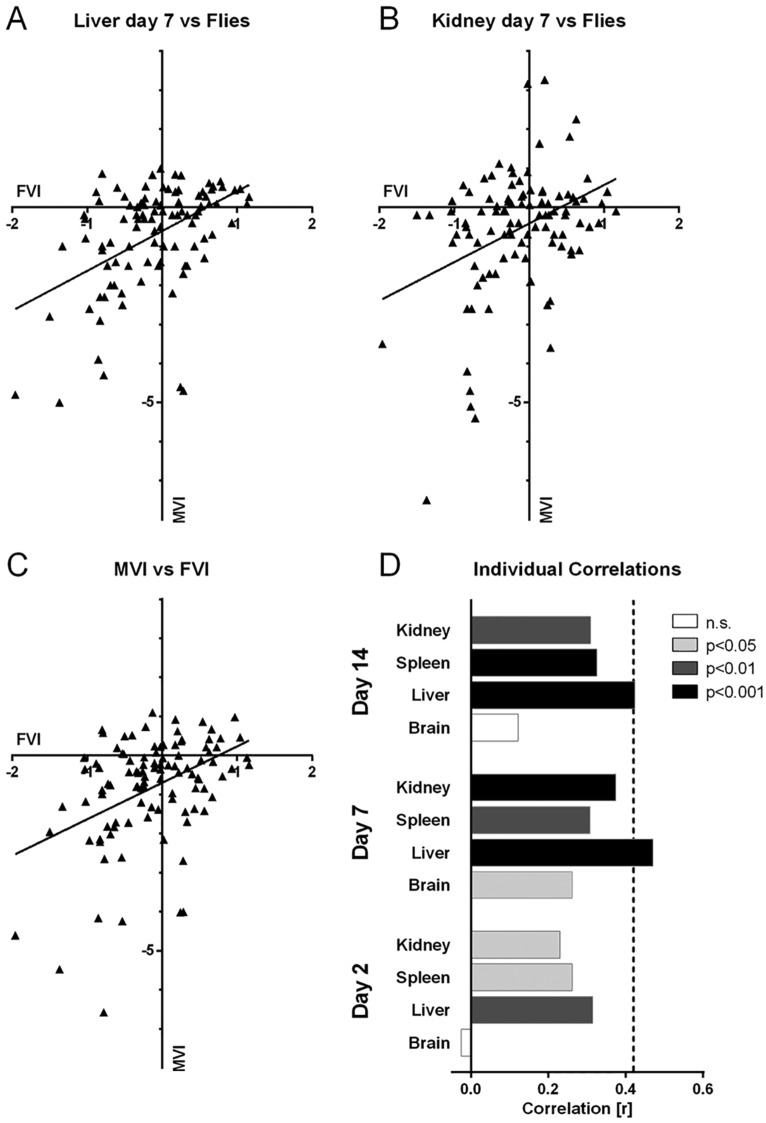
**Correlation between FVI and MVI in different organs over time.** (A-C) Correlation plots between MVI of different organs at day 7 p.i. (A,B) or the total MVI (C) against the FVI. Triangles represent plots of FVI versus MVI of each of the mutants tested in mice. The linear regression line is shown in each graph. (D) Pearson *r* values for correlations between the FVI and different organs at each time point tested. The murine liver data fit best to the *Drosophila* model. Shades of gray indicate *P* value thresholds of the individual correlations and the dotted line shows the correlation of the total MVI with the FVI.

We went on to analyze how far the data obtained from the fly model can predict the outcome of the murine model experiments. In a first step, we calculated the correlation coefficients between the FVI and the MVI of individual organs and time points (Fig. 4A-C). As can be seen in Fig. 4D, the degree of correlation depends both on the organ and the time post infection. Reasonable and statistically significant correlation was found between fly and mice data for day 7 p.i. in the target organs liver (*r*=0.47, *P*<0.001) and kidney (*r*=0.37, *P*<0.001) (Fig. 4A,B), whereas in general the brain organ burden showed poorer correlation with the fly survival rate. Earlier or later time points than day 7 generally, but not always, showed less correlation between the individual organ and the fly data. We found essentially no correlation between the fungal burden in the murine brain at day 2 p.i. and the fly survival time for our mutants (Fig. 4D).

We calculated the total MVI (the mean over all organs – excluding the brain – and all time points) for every mutant as a crude and very basic measure for total fitness of the mutant relative to the wild type over the whole course of our experimental infection. This MVI had a reasonable correlation with the fly survival times of *r*=0.42 (indicated as dashed line in Fig. 4D). Overall, the predictive value of fly survival time for the fungal burden of any given mutant is, therefore, best for the target organ liver at 7 days after infection.

**Fig. 5.**
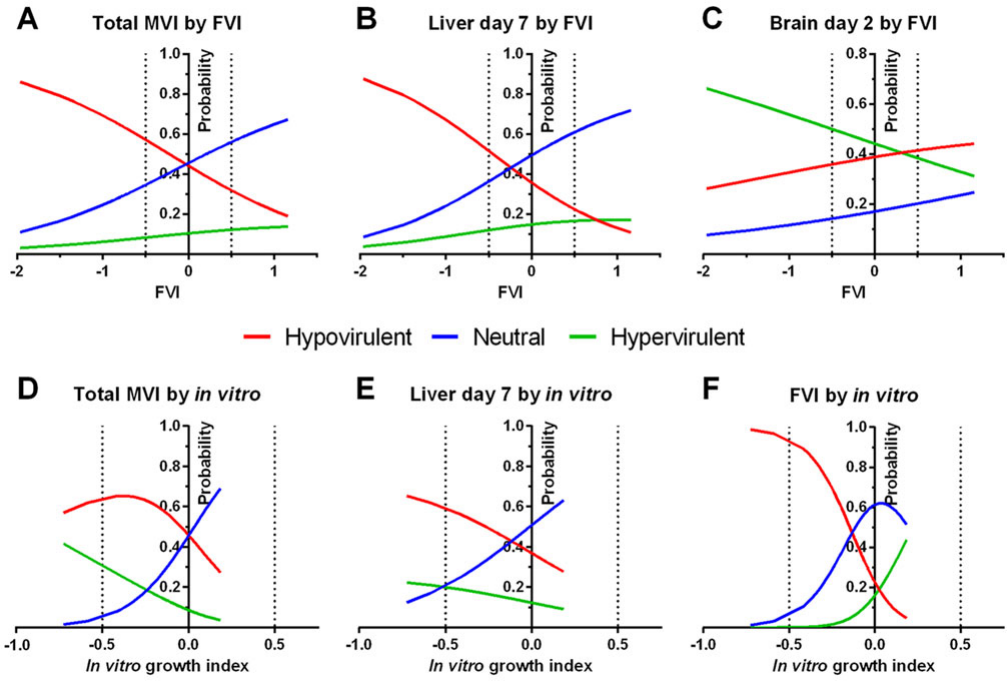
**Prediction of mouse and fly virulence classes using the fly virulence index (FVI) or *in vitro* data.** Virulence classes are referred to as hypovirulent (MVI/FVI<−0.5), neutral (−0.5≤MVI/FVI≤0.5) or hypervirulent (MVI/FVI>0.5). Multinomial logistic regression gives the probability for each mutant to fall into any of the three classes. Hypovirulence in mouse is well predicted by low FVI values, whereas hypervirulence is not. (A) FVI predicts the virulence class over all organs except for the brain (total MVI). (B) The fly model best predicts the virulence class of the mutant in the murine liver at day 7 p.i. (C) Depletion or enrichment in the brain cannot be predicted well by the FVI. Dotted lines indicate hypo- and hypervirulence in the flies based on the FVI (−0.5 and 0.5, respectively). (D,E) *In vitro* growth is not as good a predictor of the mouse virulence class as the FVI: compared with A and B, negative indices predict hypovirulence less reliably. (F) The fly virulence class is predicted well by the *in vitro* growth rate of *C. glabrata*. See also Table 3 for detailed data.

As a final and biologically relevant measure, we wanted to investigate whether virulence data obtained from the fly model is able to predict the overall virulence phenotype in mice. To this end, we simplified the MVI to three classes: hypovirulent (MVI<−0.5), neutral (MVI −0.5-0.5) or hypervirulent (MVI>0.5). We employed a multinomial logistic regression model (see Material and Methods) to use the FVI as a predictor for the mouse virulence class of the mutants. With the classes defined by the total MVI, we obtained a good predictive value of the FVI for differentiating the hypovirulence and neutral class (*P*<0.01, Table 3 and Fig. 5A). The coefficient for the hypovirulence-to-neutral class transition was −1.07, i.e. an increase of the FVI by one unit decreased the log odds ratio of the mutant to be hypovirulent versus neutral in mice organs by 1.07 (Table 3). For example, an FVI of −2 in a mutant corresponds to a 86% probability of hypovirulence and 11% of neutral behavior in mice (Fig. 5A); at an FVI of −1, these probabilities are changed to 70% and 24%, respectively. Overall, a low FVI, therefore, predicts a high probability of any mutant to be depleted in mice organ colonization.

**Table 3.**
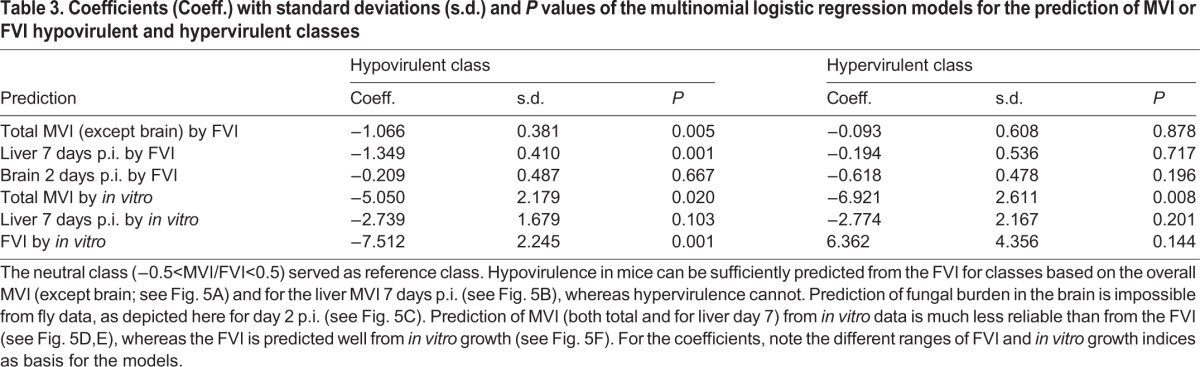
**Coefficients (Coeff.) with standard deviations (s.d.) and *P* values of the multinomial logistic regression models for the prediction of MVI or FVI hypovirulent and hypervirulent classes**

Interestingly, the neutral class becomes the most accurately predicted (>45%) only for mutants with an FVI of zero or more. In this case, an MVI virulence class on the basis of the liver data from day 7 p.i. provides better fitted results. Based on this comparison of liver data against FVI, changes in the FVI also predict well the difference between hypovirulent and neutral mutants (coefficient −1.35, *P*=0.001), but an FVI of more than −0.25 already predicts the neutral class in mice as most probable (Fig. 5B). As a control, the same model was generated with brain data from mice 2 days p.i., which did not correlate well with the FVI. As expected, the coefficient was very low (−0.2) and not statistically significant (Table 3, Fig. 5C). Similarly, completely randomized FVI-MVI pairs held no measurable predictive value (data not shown). Overall, the fly virulence index had the highest predictive power for mouse liver burden at day 7 p.i.

Interestingly, using the *in vitro* growth as the basis, the prediction of the murine virulence class is not as reliable. For the total MVI (excluding the brain), a slow *in vitro* growth predicts a hypovirulent mutant with a maximum probability of 60%, and hypervirulence is estimated to have a 40% chance (Fig. 5D) – compared with 86% and 3% probability, respectively, with the FVI as a predictor. Only slightly better results were obtained for liver at day 7 post infection (Fig. 5E). Again, the prediction of virulence class in the mouse model by using the FVI outperforms the *in vitro* data considerably (see also Table 3). Strikingly, the prediction of the FVI class itself by the *in vitro* growth index is good (with an index <−0.5 reliably predicting hypovirulence; Fig. 5F) and statistically significant, at least for hypovirulence in flies (Table 3). Hence, *in vitro* growth can reliably predict the virulence class of a given mutant in flies but not mice. To predict murine virulence classes, the FVI is much better suited.

For all hypervirulent mutants, good prediction of murine virulence from the FVI seems impossible. Whereas the probability of any mutant to be hypervirulent in mice (total MVI>0.5) rises with increasing FVI, it never exceeds ∼13% (at FVI=1, Fig. 5A) and the mutants would be classified as likely to be neutral. For high FVIs, the fly model, thus, cannot well predict the virulence class of any mutant in mice. Similar data were found for the model with the murine liver at day 7 as the data basis. Even in the *Drosophila* model, predicting hypervirulence from increased *in vitro* growth of mutants is not immediately possible, although the fastest growing mutants can reach a >40% probability of causing hypervirulence in flies (Fig. 5F).

### Individual differences of mutants between mice and flies

Overall, a decrease in FVI, therefore, is likely to indicate a defect in the murine infection model for the mutant. Looking at results from the mutants that showed large deviations between virulence indices in mouse and fly can give insight into differences between the models, as experienced by the fungus during the infection process. Here, for example, strong differences are evident in parts of the oxidative stress response. In flies, deletion of the genes encoding the main catalase (*CTA1*), the regulator of oxidative stress response (*MSN2*) or the copper transporter necessary for function of superoxide dismutases (*CTR2*) increases the FVI slightly to severely. In contrast, deleting those genes had little (*CTA1*, *CTR2*) or a severely negative (*MSN2*) effect on the fungal burden in mice.

Genes involved in cell wall integrity were found to be relevant both in flies and mice. Interestingly, genes that might be relevant for polarized growth and cell separation (*SLM1*, *MNN10*, *SLA1*, *CHS1*, *CDC12*) often – but not always – showed stronger defects in mice than in flies (supplementary material Table S3). Additionally, deletions of genes involved in later steps of outer chain protein N-glycosylation, such as *MNS1*, *MNN4* or *GNT1*, also led to strong differences between the models, with moderate to strong attenuation in mice, and an unchanged or increased virulence in flies. All these deletions led to little to no *in vitro* growth defects (Schwarzmüller et al., 2014). The deletion mutant strains of *MNN10*, *ANP1* and *VIG9*, involved in earlier synthesis steps of the mannan backbone for N-glycosylation, showed both reduced FVI and MVI.

Yet, two of these mutants, *mnn10*Δ and *vig9*Δ also had severe *in vitro* growth defects (supplementary material Table S3; Schwarzmüller et al., 2014), and the same may be assumed for *anp1*Δ as Anp1 forms a complex with Mnn10 in *S. cerevisiae* (Jungmann et al., 1999).

Finally, one arm of the HOG pathway is disrupted in *C. glabrata* ATCC 2001 (Gregori et al., 2007). In the remaining functional part of the HOG pathway, the deletion of both *SHO1* and *PBS2* led to reduction in fly and mice virulence (Fig. 1C). A deletion mutant of the Ssk1 kinase (which is part of the non-functional arm of the HOG-signaling pathway) has an FVI of −0.51 (similar to the other HOG pathway mutants) but a neutral MVI of 0.04.

## DISCUSSION

No infection model perfectly mimics human infection, and choosing the optimal model for the biological question at hand needs deliberate consideration of each model's advantages and disadvantages. Reproducibility, ease of setup and evolutionary distance to humans, as well as cost-effectiveness and ethical considerations must be taken into account (Maccallum, 2012). In many cases, it is preferable to use a simple and accessible system for a first large-scale screening. A detailed investigation of the strains found in the first test can then be performed in the more complex infection model. However, in many cases it is unclear how far the invertebrate (or simple vertebrate) model predicts the strains' behavior in a complex vertebrate model that is more similar to humans. Comparisons between models are often limited to a limited number of strains. Examples include the use of more than 30 *Pseudomonas aeruginosa* strains in *G. mellonella* (Jander et al., 2000), six *Aspergillus fumigatus* mutants (Chamilos et al., 2010) or 13 strains of *C. albicans* in *D. melanogaster* (Glittenberg et al., 2011), and 15 deletion mutants in embryonated chicken eggs (Jacobsen et al., 2011). Generally, a fair to good correlation was found between the simpler model and the respective murine model.

Our use of several hundred mutants in the fly, of which more than 100 were tested in mice, allows a good comparison of the *D. melanogaster* and *M. musculus* infection models. Similarly, a very recent study by Desalermos et al. screened 1201 *C. neoformans* deletion strains in a variation of invertebrate hosts, resulting in 12 strains that were further tested individually in mice (Desalermos et al., 2015).

### The fruit fly as a model host

*C. glabrata* generally shows a lower virulence when compared with *C. albicans*, as observed in mice (Brieland et al., 2001; Jacobsen et al., 2010a) and in embryonated chicken eggs (Jacobsen et al., 2011). In agreement with this lower virulence across different model organisms, fully immunocompetent flies do not succumb to *C. glabrata* injection (Quintin et al., 2013). Hence, we used the Toll-pathway-deficient (*MyD88*) *Drosophila* model.

Virulence of *C. glabrata* mutants with strongly reduced *in vitro* growth was often decreased in flies, as compared with the wild type. Accordingly, our model can predict the virulence class of the fly from *in vitro* growth with a good degree of confidence. However, some mutant strains with reduced FVI did not show any *in vitro* defects. Deletion of *SSD1* or that of the kinase gene *PKH1* had no effect on *C. glabrata* growth in liquid medium (Schwarzmüller et al., 2014) but reduced virulence in flies severely. Hence, in the immunodeficient fly model, growth in complex medium was a good indicator, but not sufficient to fully predict disease and death in flies. Most probably, certain virulence-associated genes are specific for fitness in the host and have little or no function *in vitro*. Moreover, temperature-related phenotypes may be impossible to detect when using flies. A possible example are calcineurin pathway genes that are involved in thermotolerance (Chen et al., 2012): *C. glabrata* deletion mutants of *CNB1*, *RCN1* and *CRZ1* were only slightly reduced in fly virulence and, hence, were not included in the murine pools. However, *CNB1* and *CRZ1* are known to be required for full virulence of *C. glabrata* in mice (whereas a *rcn1*Δ *C. glabrata* strain does not have a discernible virulence defect) (Chen et al., 2012).

Our data show the importance of an intact cell wall for the *C. glabrata* infection process in *D. melanogaster*. Many genes involved in the maintenance of cell wall integrity, either through signaling or biosynthesis of cell wall components, yielded – when mutated – the most strongly attenuated mutant strains. An interesting example are the attenuated mutant strains of the 1,3-β-glucan synthase genes *FKS1* and *FKS3*. Mutations in *FKS1* are associated with echinocandin resistance in many fungi. In *C. albicans*, however, these mutations also reduce lethality in Toll-deficient *Drosophila* (Ben-Ami et al., 2011). As *FKS1* of *C. glabrata*, but not of *C. albicans*, has been described as being functionally redundant with *FKS2 in vitro* (Katiyar et al., 2012), the reduced virulence of *C. glabrata FKS2* in *Drosophila* was surprising. This hints toward a possible non-redundant role of *FKS1* and *FKS2* (and *FKS3*) of *C. glabrata in vivo*.

Interestingly, the regulator of cell wall integrity Cas5 was found to be important in both fruit flies and a murine model of *C. albicans* infections (Chamilos et al., 2009). In fact, Cas5 was the only of 34 tested transcription factors found to be important in Toll-mutant *Drosophila*. It is also important for *C. elegans* infections (Pukkila-Worley et al., 2009). Although we did not test the *C. glabrata* ortholog of Cas5, the data indicate a similar central role of a fully intact cell wall in *C. glabrata* during infections of fruit flies. It is possible that the absence of a functional Sln1-Ssk2 arm of the HOG pathway in the ATCC 2001 strain exacerbated defects due to deletions of cell-wall-related genes: the *SHO1* arm of the HOG pathway has been implicated in cell wall integrity in *C. albicans* (Roman et al., 2005), and a deletion of *SHO1* renders *C. glabrata* hypersensitive to cell wall stress (Schwarzmüller et al., 2014). If this were the case, it would further underline the relevance of cell wall integrity in *Drosophila* infections, especially in comparison to mice.

### The murine pool infection model

Testing mutants from large-scale deletion libraries as pools of dozens of strains has been successful for several fungal pathogens in the past. Pools of ten *C. albicans* transcription factor mutants were tested, for example, by Vandeputte et al. (2011), pools of 48 *C. albicans* mutants by Noble et al. (2010), and pools of 48 *Cryptococcus neoformans* mutants each by Liu et al. for 1201 strains in total (Liu et al., 2008). Here, we have successfully used a pooled approach with microarray-based detection for a murine *C. glabrata* infection model. We validated our system by using qPCR of selected mutants and found a very good agreement, with – for unknown reason – the exception of the kidneys. Overall, however, microarray data from the kidney agreed well with spleen and liver (supplementary material Table S3), suggesting a similar behavior of most mutants in these three organs. Remaining differences can be explained by the specific conditions encountered in the organs, ranging from overall geometry, nutrient supply and presence of organ-specific host molecules to differences in immune cell populations and cytokine profiles. The latter is known to play an important role, for example, in establishing *C. albicans* infection of the kidneys (Lionakis et al., 2013). As expected by the low re-isolation counts, the brain data deviated strongly from those of the other organs. Hence, we calculated the MVI by combining data only from liver, spleen and kidney.

Our results show that the overall disease progression of pool-infected mice was not different from established single-strain infection models (Jacobsen et al., 2010a). The pool size of approximately 40 strains was chosen to keep the total number of mice in the experiment low, while enabling us to detect larger changes in mutant abundance. Smaller pool sizes would likely provide a better resolution for measuring depletion or enrichment of individual mutants, and alleviate some of the problems caused by presumed population bottlenecks in the brain. However, a reduction in pool size would also increase the number of mice needed per experiment, and fluctuations due to population bottleneck remain a problem even with pools consisting only of a few strains (Jacobsen et al., 2010a). With 40 strains per pool, we were able to detect the vast majority of mutants using microarrays. As expected, the mutants were generally depleted compared with the wild type. The most strongly depleted strains were often, but not always, mutants with a strongly reduced *in vitro* growth (Schwarzmüller et al., 2014). On the one hand, this validates our system, as we expected deletion mutants of genes such as *PFK1* to be severely reduced in growth *in vivo*. On the other hand, even mutants strongly deficient in *in vitro* proliferation, such as *ckb2*Δ, were not necessarily reduced in virulence, and many mutant strains with low fly and mice virulence were unchanged in their *in vitro* growth (supplementary material Fig. S2). Finally, mutant strains with known defects in murine virulence, e.g. a mutant lacking *YPS7*, also consistently showed up in our pool screens. The surprising finding of Cuellar-Cruz et al*.* that *CTA1* is not required for murine virulence (Cuellar-Cruz et al., 2008) was likewise evident from our data.

In summary, we were able to establish a pool infection model for *C. glabrata* mutants in mice, and to show that our data are generally in good agreement with previous studies that used single mutants. Remaining differences may be due to inherent limitations of pool experiments, like the possible *in trans* complementation of virulence defects through co-infecting strains. This can be resolved in future experiments by using mutants of interest for single-strain infections. In our model, the liver showed a high fungal burden and a consistent decrease of the burden over time, and a low inter-individual variation at early and mid-time-points, making it the most robust target organ for barcode-based *in vivo* detection of *C. glabrata* pools. This model, therefore, seems highly suitable for future applications in competitive screening of *C. glabrata* mutants.

### Correlation between mice and flies

The problems in comparing the fruit fly model – in which death of the host is the read-out parameter, with the murine model – in which fitness and growth of the pathogen serves as the measure, are self-evident. For these and other reasons, the usefulness of invertebrate models is often hotly debated. Yet, for each of these infection models, the above are the biologically relevant and – equally important – measurable parameters. Furthermore, although in our model mice do not succumb to *C. glabrata* infection, a strong correlation between fungal organ burden and virulence (measured by survival time) has been shown, e.g. for *C. neoformans* mutants (Liu et al., 2008). To enable a comparison between these two models, we have introduced the virulence scores. In mouse, an increase or decrease by one MVI unit correlates to a twofold increase or decrease in the relative *in vivo* growth of the mutant compared with wild type. In fly, a change by one on our FVI scale equals a twofold shorter or longer mean survival of the host after infection with a mutant strain, again compared with wild type. We think this represents the best combination of a biologically relevant outcome and an experimentally accessible readout for each model. Alternative approaches, such as the fungal burden in immunocompetent *D. melanogaster*, are much more difficult to quantify and, in this case, would not be as good a virulence indicator as fly death. In contrast, monitoring mouse mortality, for instance, by using specialized immunocompromised models (e.g. Atanasova et al., 2013; Ju et al., 2002), cannot be used for pool experiments as the death of the host would negate any competitive advantages due to relative fitness of the strains. For these reasons, we employed the two models and indices described above. We set the cut-offs for hypo- and hypervirulence in both models to ±0.5, equivalent to a 1.4-fold difference compared with wild type. Other, e.g. more stringent, cut-off values are feasible, depending on the type of analysis planned. In our experiment, we considered 0.5 a good cut-off that is likely to signify biological significance.

The absolute values of the correlation coefficient in our experiments differed strongly depending on the murine organ under investigation. However, given the limitations in comparing these highly different systems, the correlation between our two models strikes us as remarkably informative. Interestingly, the correlation between fly and mice virulence scores is better than between *in vitro* growth (Schwarzmüller et al., 2014) and the relative mice organ burden, which is in agreement with the multinomial logistic regression model. It is possible that different, more host-related *in vitro* growth conditions (such as nutrient-poor or less oxygenated conditions) will result in a better agreement with *in vivo* fitness. However, the changing conditions and defences encountered in the host are unlikely to be simulated within any *in vitro* system, whereas the fruit fly model presents many of the stresses encountered in mammals.

The multinomial logistic regression model itself was highly informative. By reducing murine fitness and virulence potential to the three classes that are most interesting to the researcher, viz. hypovirulent, hypervirulent or neutral with respect to the wild type, we were able to provide a suitable predictive model. With our data it is possible to estimate the probability of any mutant to be reduced in murine fungal burden, based on a previous test using the fly model. The log of the odds ratio, i.e. the probability for being hypovirulent divided by the probability for being neutral, decreases quickly with increasing FVI. From the data in Fig. 5, it is clear that a low FVI (and, thus, a long mean survival time of flies) gives a high degree of confidence in predicting the mutant to be depleted in mice organs.

*In vitro* growth per se is often considered a good predictor of microbial burden *in vivo* (see e.g. Paisley et al., 2005), as growth is a prerequisite for organ colonization. However, in our multinomial logistic regression models, the virulence class of the mutants within the mouse was not well predicted by *in vitro* growth alone. This is reflected by the relative abundance of mutants with reduced virulence indices and unaltered *in vitro* growth. Similar discrepancies have previously been found, e.g. in large-scale mutant libraries of *C. albicans* (Noble et al., 2010) and *C. neoformans* (Liu et al., 2008), where most mutants depleted in pooled murine infection models displayed normal *in vitro* growth. Accordingly, *in vitro* growth alone seems insufficient to fully predict fitness in the mouse. Hence, the fly model (through the FVI) is superior to the simple *in vitro* growth assays and can be used to predict fitness and virulence potential of *C. glabrata* mutants in the mouse. This better correlation of mice and fly data – compared with those of *in vitro* growth – indicates the existence of additional selection pressures that are specific for fungal growth in host organisms. Use of the simple fly model should, therefore, allow a better detection of bona fide virulence factors.

### Specific differences and similarities

The differences observed between the models for some of the tested mutants may be informative with respect to the hosts' differing responses. On the one hand, several aspects of immunity, such as the adaptive response and certain kinds of innate immune cell, e.g. dendritic and natural killer cells, do not exist in *Drosophila*. The melanization reaction of *Drosophila*, on the other hand, has no counterpart in mammals, although it cannot be activated in Toll-pathway mutants (Ligoxygakis et al., 2002). Certain aspects of interaction between the fungus and the immune system might, therefore, differ substantially between the models, especially when – necessarily – using Toll-cascade-deficient flies.

So far, for example, it is unknown whether *Drosophila* hemocytes use an oxidative burst to kill ingested fungi. Whereas *C. glabrata* is known to be phagocytosed by hemocytes (Quintin et al., 2013), we found that a strain harboring a deletion of the single catalase gene *CTA1* in *C. glabrata* (Cuellar-Cruz et al., 2008) kills the flies even faster than the wild type. The same is true for Msn2, a transcription factor involved in the general stress response including oxidative stress (Cuellar-Cruz et al., 2008), and Ctr1, a Cu^2+^ transporter required for full superoxide dismutase activity. This suggests that these antioxidant activities are either highly redundant (Briones-Martin-Del-Campo et al., 2014) or not required for survival in our *Drosophila* model *in vivo*. Previously (Quintin et al., 2013), we have asked whether the *C. glabrata* catalase plays a role in withstanding the cellular immune response in *Drosophila*. The data presented here at least indicate that this is not the case because phagocytosis of *C. glabrata* by hemocytes takes place in our *Drosophila* model (Quintin et al., 2013), but the oxidative stress response seems dispensable for fungal virulence during fly infections. For the catalase Cta1, our findings confirm previous murine systemic infection experiments with *C. glabrata* where Cta1 also did not play a role (Cuellar-Cruz et al., 2008, and shown here). In contrast, however, our data indicate an important role of the transcriptional regulator Msn2 in murine infections.

An interesting aspect is the role of the HOG pathway in infections. *C. glabrata* ATCC 2001 is lacking one of the two branches of this osmotic stress resistance system and, therefore, relies solely on Sho1-Pbs2 signaling for osmoprotection (Gregori et al., 2007). Hence, the HOG pathway is completely disrupted in the *sho1*Δ and the *pbs2*Δ mutant, and both were found to be decreased in fly virulence. Whereas the *pbs2*Δ *C. glabrata* strain was not tested in the mouse model, we found the *sho1*Δ strain to be also strongly depleted in mice. In addition, we recently found *sho1*Δ *C. glabrata* to be more susceptible to killing by human macrophages (Seider et al., 2014). This indicates that the sole remaining HOG pathway is necessary for full fly and mice virulence in *C. glabrata*, and is most likely to be relevant in (human) phagocyte-pathogen interactions. The HOG pathway is also of importance in *C. albicans* infections (Alonso-Monge et al., 1999), where Sho1 plays a role in sensing oxidative rather than osmotic stress. The latter function is not present in *C. glabrata* Sho1 (Gregori et al., 2007), which fits well with our observation that the catalase Cta1 was not required for full virulence. Finally, the Ssk1 kinase of the disrupted Sln1-dependent arm of the HOG pathway seems to still play a role in *Drosophila*, but not in murine infections with *C. glabrata* ATCC 2001.

Interestingly, many glycosylation-deficient mutants appeared either in both our systems or, specifically in mice only, as reduced in virulence. Early steps in N-glycosylation seem to have a huge effect in interaction with both hosts, showing the importance of glycosylation per se. Differences between the hosts appear with mutants of later steps in glycosylation and are, thus, likely to be due to different interactions between specifically glycosylated fungal proteins and their respective host receptors. The *och1*Δ mutant of *C. albicans*, defective in the initiation of the outer chain N-glycosylation, was described as hypovirulent in mice (Bates et al., 2006), but the homolog was not part of our tests. Interestingly, one of the strongly reduced *C. glabrata* mutants in mice (and to a certain extent flies) – *mnn10*Δ – has recently been found to be important for survival in macrophages and has been implicated in active alkalization of the phagosome (Kasper et al., 2014).

### Conclusions

Animal experiments should always be guided by the principles of refinement, reduction and replacement (3R). We have defined two new measures for virulence in flies and mice, and established the corresponding methods to determine them. In mice, we were able to demonstrate the feasibility of using pools of up to 40 barcoded *C. glabrata* mutants to determine fitness and virulence potential in murine infections. We have also shown that, for *C. glabrata* and probably other pathogenic microbes, fruit flies are a suitable model to predict the outcome of murine infections, especially following infection with severely attenuated mutants. Using our approach, a pre-screening of mutants in the invertebrate model *Drosophila* provides a good estimate of the probability to find a reduced microbial burden in mouse host with the same mutant. This pre-screen can be especially useful for selecting mutants from large, systematic genome-wide collections of deletion strains for validation in the mouse model, using a minimum number of mice. Additionally, this system is of potential interest for microbial drug target screens. *Drosophila* infections were superior to *in vitro* growth assays in predicting reduced fitness of mutants in mice. The corresponding gene products, therefore, include putative drug targets that would not have been selected by *in vitro* growth assays alone but play important roles within the host. This seems especially relevant in the light of previous landmark papers on large-scale, pool-based screens of *C. albicans* and *C. neoformans* mutant libraries in mice (Liu et al., 2008; Noble et al., 2010). In these studies, there was a good general agreement between *in vitro* growth defects of mutants and decreased presence in target organs. The authors also found many mutants whose presence was depleted in organs despite their wild-type-like *in vitro* growth; screening of an alternative infection model may have detected many of these strains. Desalermos et al. recently added an interesting twist to this approach by suggesting to use a range of different invertebrate infection models to best cover different mechanisms of virulence (Desalermos et al., 2015).

The remaining differences between the fruit fly and murine models must be viewed in light of the system used. Both systems have their specific limitations and do not fully reflect infection in humans; and no decision can easily be made as to which mutant phenotype is ‘more real’ or ‘better’ than the other when the two models give different results. Even the canonical murine models do not fully reflect human infections: for example, mice have a different commensal microbiome, and typical risk factors in humans include compromised immunity, underlying diseases and old age, which are not represented in the murine model. With these limitations in mind, functional insight into the infection process can be gained especially by comparing the data from the models. Clearly, using the ‘simpler’ model for a preliminary screen is preferable for ethical, financial and practical reasons. These first models are not limited to *Drosophila* but can include, for example, *C. elegans* (Breger et al., 2007), *G. mellonella* (Borghi et al., 2014; Jacobsen, 2014), zebrafish (Gratacap and Wheeler, 2014) or embryonated eggs (Jacobsen et al., 2011). It is especially noteworthy that these simple models allow better predictions of murine organ burden than an *in vitro* growth assay. Virulence factors common to the simple and the complex model that, nonetheless, have no discernible *in vitro* phenotype can, thus, be detected more reliably.

## MATERIALS AND METHODS

### Strains and growth conditions

The deletion mutants are described elsewhere (Schwarzmüller et al., 2014). Briefly, all mutants are based on a triple-auxotrophic (*trp1*Δ, *leu2*Δ, *his3*Δ) derivative of the *C. glabrata* ATCC 2001 reference strain. Genes were replaced by a *NAT1* marker cassette with one of 96 different genetic barcodes (Schwarzmüller et al., 2014). The reference wild type contains its own specific barcode. All strains were routinely grown in YPD complex medium at 30°C, 180 rpm.

### *D. melanogaster* infection model

*D. melanogaster* survival experiments were performed as previously described (Quintin et al., 2013). Briefly, batches of 20-25 *MyD88* mutant flies (Tauszig-Delamasure et al., 2002) were challenged by septic injury using a needle dipped into a concentrated solution of *C. glabrata*, or using a thin capillary filled with yeast cells resuspended in PBS (OD_600_=20) containing 0.01% Tween to avoid agglutination. The thorax was injected with 9.2 nl using a Nanoject II apparatus (Drummond Scientific, Broomall, PA). Vials containing infected flies were put in an incubator at 29°C and surviving flies counted every day. Flies were moved into fresh vials every other day.

To calculate the fly virulence index (FVI), data on fly death rates were fitted to Weibull distributions as suggested for *C. albicans* (Glittenberg et al., 2011), and the time at which 50% of the animals had succumbed to the infection was calculated (LT_50_). For each infection group, a reference wild type was included. The FVI is the log_2_ of the LT_50_ ratio of mutant and corresponding wild type.

### Pool composition and murine infections

On the basis of the *D. melanogaster* data, four randomized pools were composed comprising strongly attenuated, moderately attenuated, hypervirulent, and neutral control strains, plus the barcoded wild type (supplementary material Table S2). Strains were precultured individually overnight and mixed in equal amounts by their OD_600_. The pooled yeasts were washed twice with PBS and adjusted to 2.5×10^8^ cfu/ml for infection.

Specific-pathogen-free, outbred, female, 5-week-old CD-1 mice weighing 18-22 g (Charles River, Germany) were housed in groups of five in individually ventilated cages and cared for according the principles outlined in the *European Convention for the Protection of Vertebrate Animals Used for Experimental and Other Scientific Purposes*. All animal experiments were in compliance with the German animal protection law and approved by the responsible Federal State authority and ethics committee (permit no. 03-008/07). For infection experiments, mice were intravenously challenged with 5×10^7^ cfu in 200 µl PBS, and their health status was subsequently examined twice daily by a veterinarian. On days 2, 7 and 14 post infection, three animals per tested pool were sacrificed (Jacobsen et al., 2010a). Post mortem analysis included recording of macroscopical changes and determination of fungal burden. Kidneys, spleen, liver and brain were removed aseptically at necropsy, rinsed with sterile PBS, weighed and aseptically homogenized in PBS using an IKA T10 basic Ultra-Turrax (Ika, Staufen, Germany).

As the number of fungi inside organs was too small for direct isolation of DNA, homogenized organs and the original infection pool were plated and grown for 28 h. Plates with a maximum of 2000 colonies were used for DNA extraction after scraping in PBS (Sambrook and Russell, 2001). For nearly all organs and time points, sufficient colonies (i.e. hundreds) grew, except for in the brain at later time points. Data from these few colonies were initially included in the analyses, with the caveat that they might be unreliable due to the low sampling numbers.

### Array design and barcode detection

Arrays were designed to detect the barcodes. Isolated fungal DNA was mixed with DNA standards containing additional barcodes. Barcodes were PCR-amplified and labeled with cyanine dyes. DNA isolated from the plated inoculation pools and from the re-isolated strains (pooled from three mice each) were labeled with different cyanine dyes for a two-color hybridization with dye swaps to obtain two technical replicates. Array data were evaluated using GeneSpring GX, Version 12.1 (Agilent, Santa Clara, CA). Only data with signal-to-noise ratios of >2 in the inoculum were included. The relative amount of a mutant following infection was determined by dividing the signal intensities of the mutant's barcode in the inoculum by the signal intensity in the re-isolated pool. Enrichment or depletion of the mutant was calculated by dividing this ratio by the ratio obtained for the wild type for the same sample for a relative *in vivo* fitness of the mutant. The MVI is the log_2_ transformation of that ratio (Sig=signal intensity): MVI=log_2_[(Sig_(Re-isolated|Mutant)_/Sig_(Inoculum|Mutant)_)/(Sig_(Re-isolated|WT)_/Sig_(Inoculum|WT)_)]. Only mutant strains with at least six data points (half of the total of 12 organ/time pairings) were considered for further analysis.

### qPCR measurements

Barcode-specific quantitative PCRs (qPCRs) were performed using DNA of fungal cells isolated from individual mice. Briefly, 100 ng of genomic DNA (gDNA) from plated inoculation pools or re-isolated strains was used to detect specific barcodes (Schwarzmüller et al., 2014) by using the EvaGreen QPCR Mix (Bio&Sell, Feucht, Germany) in a C1000/CFX96 Thermal Cycler (Bio-Rad, Munich, Germany). Barcode amounts were calculated by standard curves with the mutant's gDNA. At least three independent experiments were performed. Mutants with less than two measurable c_t_ values were excluded.

### Statistical analyses

GO term enrichment was calculated using GO::TermFinder (Boyle et al., 2004), with all mutants screened in the fly as background population. Sets of genes were considered enriched if *P*<0.05 and the false discovery rate Q<5%. GO terms were obtained from the Candida Genome Database (www.candidagenome.org). For correlation analyses, GraphPad Prism, version 6.00 (GraphPad Inc., La Jolla, CA) was used. For correlation analysis of organ means, mouse data were only used when values for >50% of organs and time points were available. Correlation values are given as Pearson correlation coefficient *r*.

For the multinomial logistic regression models, MVI and FVI were transformed into three categorical variables (virulence class), with the reference class neutral (−0.5<MVI/FVI<0.5), hypovirulent (MVI/FVI<−0.5) and hypervirulent (MVI/FVI>0.5). Predictor variables for the mouse virulence class were FVI values or previously published *in vitro* data – the relative growth in YPD medium at 30°C, compared with the average growth rate of the whole collection (Schwarzmüller et al., 2014). For fly virulence class, log_2_ converted *in vitro* data served as predictor variables. Models were fitted and coefficients were obtained by using R software (version 2.15.2) and the nnet package (version 7.3-5).

## Supplementary Material

Supplementary Material
