# Supplementary material for: Of mice, flies – and men? Comparing fungal infection models for large-scale screening efforts
Source: Dis Model Mech. 2015 May 1;8(5):473–86. doi: 10.1242/dmm.019901 (PMC4415897; doi:10.1242/dmm.019901)
Supplement: Supplementary Material [file supp_8_5_473__index.html]

Of mice, flies – and men? Comparing fungal infection models for large-scale screening efforts — Supplementary Material 

# Of mice, flies – and men? Comparing fungal infection models for large-scale screening efforts

## DMM019901 Supplementary Material

**Files in this Data Supplement:**

- **Supplementary Material**
