# Supplementary material for: Of mice, flies – and men? Comparing fungal infection models for large-scale screening efforts
Source: Dis Model Mech. 2015 May 1;8(5):473–86. doi: 10.1242/dmm.019901 (PMC4415897; doi:10.1242/dmm.019901)
Supplement: Supplementary Material [file supp_8.5.473_DMM019901.pdf]

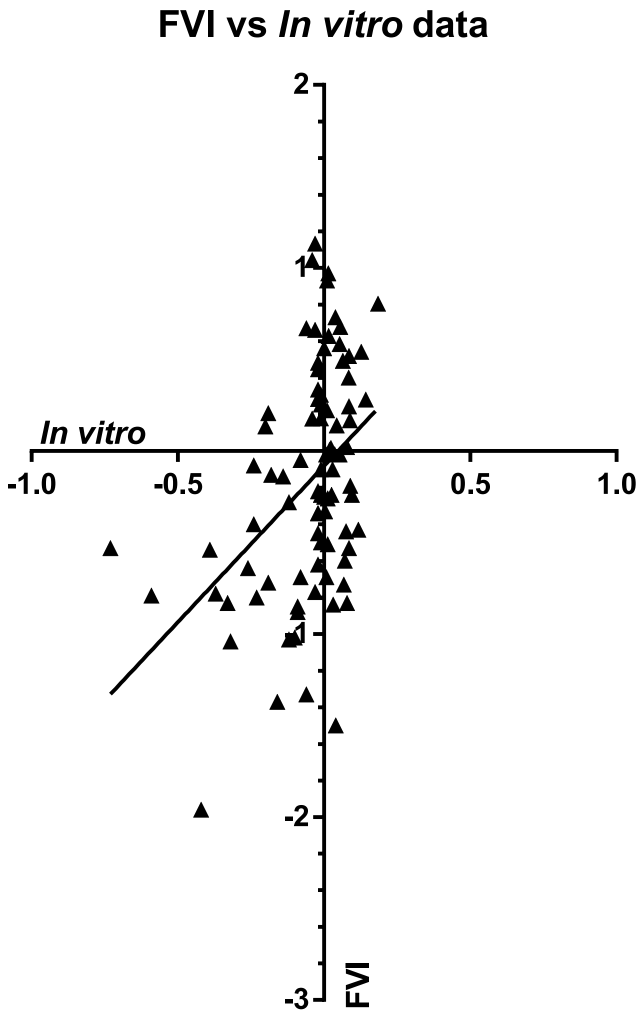

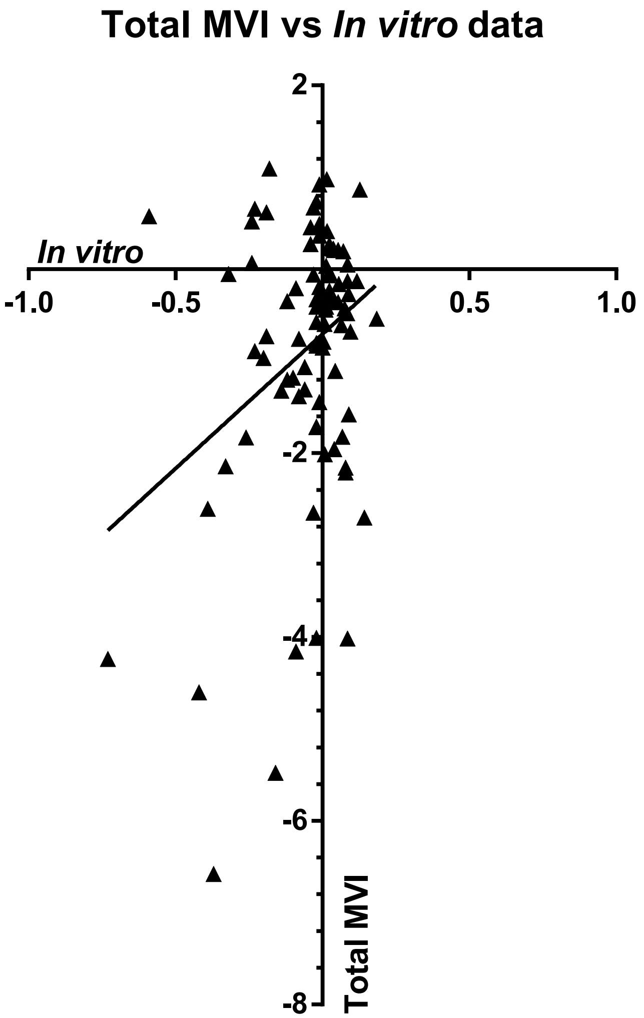

Fig. S1: Correlation of *in vitro* growth data with FVI and total MVI for all mutants included in the pools.

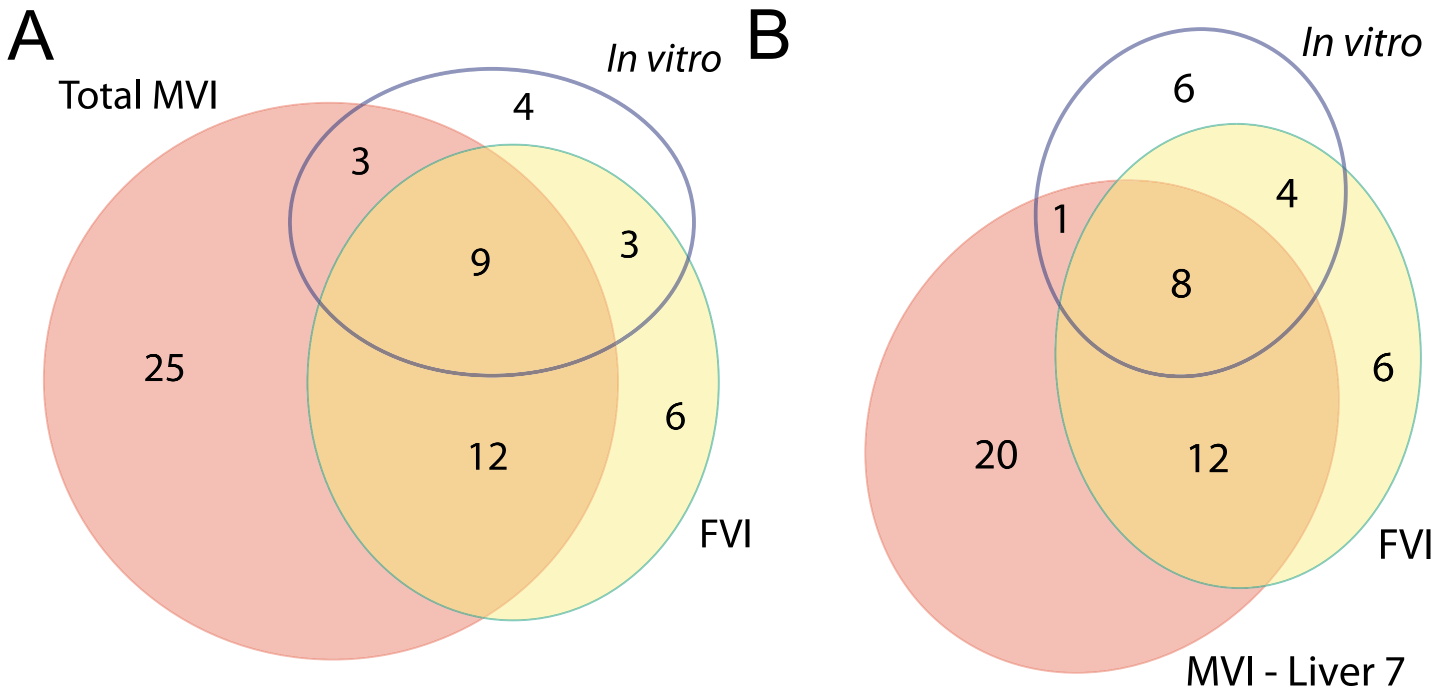

Fig. S2: Venn diagrams showing the overlap between mutants found depleted in mice (MVI<-0.5), hypovirulent in flies (FVI<-0.5) or defective in *in vitro* growth (relative fitness reduced by >2σ; see (Schwarzmüller et al., 2014)), (A) Total MVI (liver, kidney and spleen over all sampling days), (B) Liver MVI at day 7 p.i. Note that in both cases, the overlap between reduced MVI and FVI is larger than between MVI and *in vitro* growth defects

Table S1. Fly virulence indices (FVI) of all tested mutants, with gene identifiers and descriptions based on the *Candida* Genome Database.

Table S2. Composition of the four pools tested in mice with FVI values as reference and specific relevant notes for individual mutants

Table S3. Overview of individual and total MVI, FVI, and *in vitro* growth data for all mutants reisolated from mice.

Download Tables S1 - S3
